# Studies on Bioactive Components of Red Ginseng by UHPLC-MS and Its Effect on Lipid Metabolism of Type 2 Diabetes Mellitus

**DOI:** 10.3389/fnut.2022.865070

**Published:** 2022-04-27

**Authors:** Rensong Huang, Meng Zhang, Yu Tong, Yaran Teng, Hui Li, Wei Wu

**Affiliations:** Changchun University of Chinese Medicine, Jilin Ginseng Academy, Changchun, China

**Keywords:** red ginseng, UHPLC-MS, type 2 diabetes mellitus, lipid metabolism, short-chain fatty acids

## Abstract

**Objectives:**

Red ginseng is a processed product of *Panax ginseng* C.A. Meyer, which is one of the widely used medicinal and edible herbs for the treatment of type 2 diabetes mellitus (T2DM). Ginsenosides are its main pharmacologically active ingredient. This study aims to clarify the material basis of total ginsenosides of red ginseng (RGW) and verify the activity of RGW in treating lipid metabolism disorders caused by T2DM.

**Methods:**

An ultrahigh performance liquid chromatography coupled with quadrupole time of flight mass spectrometry (UHPLC-Q-TOF-MS) technology was applied to quantitatively analyze RGW. A T2DM rat model was established to verify the activity of RGW in treating lipid metabolism disorders caused by diabetes. First, the changes in diabetes-related parameters were observed, then the biochemical parameters of the rat serum and liver were measured, and finally, the pathological sections of the rat liver were observed, and the content of short-chain fatty acids in stools was measured. The *in vitro* activity of RGW was verified by fatty degenerated HepG2 cells.

**Results:**

A total of 10 ginsenosides were identified and quantitatively analyzed in RGW. Experimental results demonstrated that RGW can improve lipid metabolism disorders. RGW significantly reduced the fasting blood glucose and TG and TC levels in T2DM rats, and hepatic steatosis was significantly ameliorated. *In vitro* experiments by RGW treatment also significantly attenuated lipid deposition in HepG2 cells. RGW upregulated the content of 5 short-chain fatty acids in rat stools, which are related to lipid oxidation and liver gluconeogenesis.

**Conclusion:**

The total RGW were quantitatively analyzed by UHPLC-MS, and its effect on lipid metabolism of T2DM was studied. The experiment demonstrated that red ginseng can regulate lipid metabolism and improve lipid deposition, which provides a promising development for red ginseng as a functional food.

## Graphical Abstract

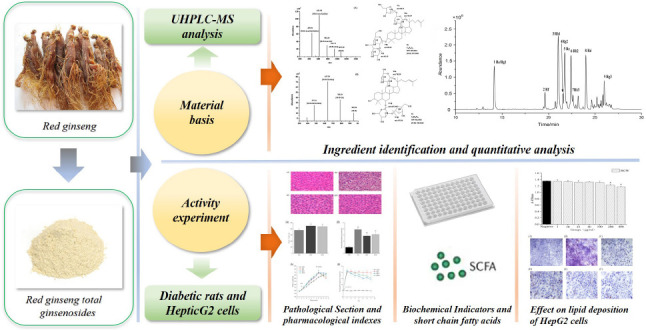

## Introduction

*Panax ginseng* C.A. Meyer was used as an herbal or tonic food in Eastern Asia for a long time (1), and recently, was approved by the Ministry of Health of the People’s Republic of China as a new food resource that can be used in the healthcare sector. Several studies have been conducted to elucidate its bioactive components as well as its pharmacological actions, considering its health-related benefits in daily life as a nutritional product. Specifically, red ginseng (Ginseng Radix et Rhizoma Rubra; RG), which has a “warming effect” and is used in Chinese medicine to “boost yang” and replenish vital essence, is a thermally processed ginseng product that is obtained by steaming fresh ginseng at a certain temperature followed by drying (2). Furthermore, RG extract is widely used in healthcare (3), and its current product forms primarily include natural roots, powder, tablets, tea, extracts, and beverages. Furthermore, the biotransformation and health benefits of ginseng and RGW, and their application in dairy products have also been reported (4). In particular, lactic acid bacteria-fermented RG, with lipid-lowering activity, has been used as a health food in China. It has also been observed that RG contains high amounts of ginsenosides, which are bioactive components with various pharmacological effects. Furthermore, studies involving diabetic animal models and cells have shown that ginsenosides exert antidiabetic effects by regulating the production and secretion of insulin and improving glucose and lipid metabolism as well as inflammation (5). Using LC-MS, ginsenosides have been identified from ginseng and its related processed products such as protopanaxatriol (PPT), protopanaxadiol (PPD), and oleanolic acid based on the structure of the aglycone skeleton (6). Additionally, these ginsenosides show a wide range of biological activities owing to their species diversity and different chemical structures. Thus, to ensure the quality, safety, and efficacy of ginseng and related products, it is often necessary to monitor the constituent active ingredients. In this regard, LC-MS/MS analysis techniques are of great significance. Studies with a focus on the overall as well as the multi-target effects of ginseng have also been conducted by monitoring the multiple bioactive components of ginseng (7, 8), and LC-MS/MS has also been successfully applied in the identification of ginsenosides in ginseng, white ginseng, and RGW extracts (9). In a previous study, non-targeted metabolomics based on UPLC-MS/MS was developed to elucidate the different mechanisms of action of American ginseng traits in human systems (10).

Type 2 diabetes mellitus, which induces a continuous increase in blood glucose levels and increases the risks associated with different syndromes (e.g., diabetic nephropathy, diabetic eye disease, and diabetic cardiomyopathy), is a chronic metabolic disease that is mainly caused by insufficient insulin secretion or insulin resistance (11, 12). In addition to abnormal glucose metabolism, McGarry reported that lipid metabolism disorders also play an important role in the development of T2DM metabolic disorders (13). Specifically, lipid metabolism disorders, which have an important relationship with various major diseases in the human body, represent one of the main classes of clinical manifestations of T2DM (14). Reportedly, obesity is a trigger for insulin resistance. This is because excessive fat accumulation can lead to abnormal glucose metabolism, which can then impair the metabolic functioning of multiple organs, including adipose tissue and the surrounding organs, thereby aggravating the disease (15). Excessive fat accumulation will also lead to abnormal changes in lipid-related biochemical indicators such as triglyceride (TG) and total cholesterol (TC). Therefore, regulating lipid metabolic disorders is of great practical significance in the prevention and treatment of T2DM. Currently, the main drugs for T2DM treatment are insulin and oral hypoglycemic agents (16, 17). They have potential side effects (18, 19). Thus, the use of natural products as alternative anti-T2DM agents has attracted extensive attention over the years. Some natural products, such as ginsenosides, have been studied for their hypoglycemic activity and effects on the treatment of diabetes (20–22). In particular, ginsenoside Rg1 protects mice against streptozotocin (STZ)-induced type 1 diabetes by modulating the NLRP3 and Keap1/Nrf2/HO-1 pathways (23). Furthermore, ginsenoside Rk3 ameliorates insulin resistance, prevents inflammation, and improves lipid accumulation and gluconeogenesis in HFD/STZ-induced T2DM mice *via* the AMPK/Akt signaling pathway (24), and ginsenoside Re can reduce blood glucose levels, increase insulin levels, improve lipid metabolism, and reduce endothelial cell dysfunction by modulating the p38 MAPK, ERK1/2, and JNK signaling pathways to produce antidiabetic effects (25). Furthermore, ginsenoside Rg3 administered before islet transplantation enhances islet cell function and attenuates cytokine-induced injury (26). Previous studies have also shown that ginsenoside Rg2 treatment can significantly reduce TG and TC levels in oleic acid and palmitic acid (OA&PA)-induced mouse primary hepatocytes, and this was also confirmed by Oil Red O staining (27). The current studies on the pharmacological activities of ginsenosides mostly focus on monomer compounds, thus, exploring the antidiabetic activity of the ginsenosides in RGW for multiple target functions can be of great significance. Studies have also shown that short-chain fatty acids (SCFAs) are closely related to diabetes in that they improve insulin resistance and pancreatic damage while attenuating the inflammatory responses caused by diabetes (28). At the same time, it can also increase the expression of G protein-coupled receptor 43 mRNA in the colon and increase the levels of hormones GLP-1 and Peptide YY, thereby repairing glucose intolerance in type 2 diabetic mice (29).

In this study, in the first place, UHPLC-Q-TOF-MS was used to quantitatively analyze the total ginsenosides in RG (RGW) for quality control. Furthermore, the effects of RGW on lipid metabolism disorders caused by T2DM from three aspects were comprehensively studied: *in vivo*, *in vitro*, and metabolites. Specifically, a T2DM rat model was established to verify the ability of RGW to treat lipid metabolism disorders caused by diabetes. Thereafter, diabetes-related parameters of rats were studied, and biochemical indicators in rat serum, as well as hepatic and short-chain fatty acid contents in stool samples from the rats, were measured. *In vitro* experiments were also performed using fatty degenerated HepG2 cells to verify the activity of RGW, and pathological sections of the liver were also observed.

## Materials and Methods

### Plant Material and Reagents

The *P. ginseng* used in this study was purchased from Ji An (Jilin, China) in June 2020 and was processed at our laboratory to obtain RG. The preparation method for RG was as follows: cleaned fresh ginseng was placed in a steaming box, the temperature was increased to 100°C within 60 min, and steaming was continued for 3 h; then, the ginseng sample was cooled and taken out. The steamed ginseng samples were dried in an oven at 50°C to obtain RG. Botanical identification was undertaken by Professor L. Jiao, and the voucher specimen (No. 20200005) was kept at the Jilin Ginseng Academy, Changchun University of Chinese Medicine, China.

The red ginseng was soaked overnight in a given volume of distilled water. Eight times the volume of distilled water was added, and the extraction was performed four times at 80°C for 1 h. The extracts obtained were combined and centrifuged. The resulting supernatant was then concentrated to the appropriate volume in a water bath at 60°C. Extraction was performed on the concentrated supernatant three times using n-butanol, and the n-butanol layer was collected. Then the n-butanol layer was evaporated to dryness, dissolved by adding an appropriate volume of DW, and freeze-dried to obtain RGW.

Furthermore, ginsenoside standards Re, Rg1, Rf, Rb1, Rg2, Rc, Rb2, Rb3, Rd, and Rg3 were purchased from Shanghai Yuanye Bio-Technology Co., Ltd. (Shanghai, China), and STZ, 2-ethylbutyric acid, acetic acid, propionic acid, butyric acid, valeric acid, and isovaleric acid were purchased from Sigma Chemical Co. (St. Louis, MO, United States). Citric acid and sodium citrate were purchased from Beijing Taibo Chemical Co., Ltd. (Beijing, China), acetonitrile and chromatographically pure methanol (MeOH) were obtained from Fisher Scientific (Waltham, MA, United States), and a Milli-Q device (Millipore, Milford, MA, United States) was used to produce ultrapure water. Furthermore, commercial reagent kits for the determination of TC, TG, high-density lipoprotein (HDL-C), and low-density lipoprotein (LDL-C) levels were obtained from Nanjing Jiancheng Biotech. Co., Ltd. (Nanjing, China), while concentrated sulfuric acid and ether were purchased from Beijing Chemical Works (Beijing, China).

### UHPLC-Q-TOF-MS Analysis

Total ginsenosides of red ginseng and the 10 reference compounds (1 mg each) were accurately weighed and dissolved in a 1-ml volumetric flask containing 80% chromatographic methanol. Thereafter, the resulting solutions were filtered through a 0.22-μm membrane filter, followed by UHPLC-MS analysis. The UHPLC-MS analysis of RGW was performed using a UHPLC-ESI-Q-TOF/MS system. Specifically, UHPLC was performed using an Agilent 1200SL RRLC system (Agilent Technologies; Waldbronn, Germany) coupled with an Agilent SB-C18 column (3.0 × 100 mm, 1.8 μm, 600 bar). The mobile phase consisted of 0.1% formic acid in water (solvent A) and acetonitrile (solvent B) eluted at 0.30 ml/min and the elution gradient was set as follows: 0–5 min, 19% B; 5–12 min, 19–28% B; 12–22 min, 28–40% B; 22–24 min, 40–85% B; 24–25 min, 85–19% B; and 25–30 min, 19% B. Each injection volume was set at 5.0 μl and the column temperature was maintained at 30°C. Mass spectrometric analysis was performed *via* Q-TOF mass spectrometry. Specifically, the electrospray ionization source in the negative (ESI-) ion mode, with a scanning range of m/z ∼200–3,000, was used. The MS source parameters were set as follows: drying gas flow rate, 10.0 L/min; vaporizer temperature, 350°C; nebulizer pressure, 255 kPa; capillary voltage, 3.5 kV; fragmentor voltage, 200 V; cone voltage, 65 V; and octopole RF voltage, 250 V. All data were acquired and analyzed using Mass Hunter Qualitative and Mass Profiler Professional (MPP, version B. 02.01, MHQ, version B.03.01, Agilent Technologies, Santa Clara, CA).

### Validation of UHPLC-MS Method

#### Calibration Curves, Limits of Detection, and Quantification

To construct calibration curves, 1 mg/ml methanol stock solutions of the 10 reference compounds were diluted to the appropriate concentrations. Thereafter, six concentrations of the different solutions were analyzed in triplicate, and calibration curves were constructed by plotting the peak areas corresponding to the extracted ion current spectra against the analyte concentrations. Furthermore, suitable concentrations were injected into the UHPLC-MS system for analysis, and the limits of determination (LOD) and the limits of quantification (LOQ) under the prevailing conditions were determined at signal-to-noise (S/N) ratios of approximately 3 and 10, respectively.

#### Precision, Accuracy, and Stability

First, a precision experiment was performed. The method was tested for inter-day and intraday precision by analyzing the same sample six times a day on three separate days. The experimental results were expressed as relative standard deviation (RSD) values. Additionally, recovery tests were used to determine the accuracy of the method. Accurate amounts of 10 ginsenosides were added to the solution of a known concentration of the test substance. The average recoveries were calculated using the following formula: recovery (%) = (amount found-original amount)/amount spiked*100%. Samples were analyzed at 0, 2, 4, 6, 8, 10, 12, 16, and 24 h using an established method for stability assessment. Thus, the stability was expressed as the RSD values corresponding to the nine data points. Six samples were used for each experiment.

### Animal Experiments

The animal experiments were approved by the Institutional Animal Care and Use Committee (IACUC) of Changchun University of Chinese Medicine (Approval number: CPCCUCM IACUC 2020-040). A total of 60 adult male Wistar rats were placed in an SPF barrier environment under standard environmental conditions (temperature, 25°C; relative humidity, 55 ± 5%) with unrestricted access to water and food. After 1 week of acclimatization, all the rats were randomly divided into four groups (*n* = 15), namely, the CON (healthy animals treated with DW), DM (T2DM animals treated with DW), MET (T2DM animals treated with metformin at 100 mg/kg/day), and RGW (T2DM animals treated with RGW at 100 mg/kg/day) groups. The CON group was fed with ordinary feed, while the other three groups were fed a high-fat diet (HFD) consisting of 18% lard, 20% sucrose, 3% egg yolk powder, and 59% pulverized normal rat feedstuff. After the HFD feeding for 8 weeks, the rats were fasted for 12 h but were free to drink. Rats in the CON group then received an intraperitoneal injection of citrate buffer, while those in the other three groups received an intraperitoneal injection of low-dose STZ solution (35 mg/kg) dissolved in sodium citrate buffer (0.1 mol/L, pH 4.3–4.5). A fasting blood glucose level ≥ 11.1 mmol/L indicated the successful establishment of the T2DM rat model. The RGW intervention process then lasted for 21 days, and the fasting glucose level in blood samples from the tail vein (approximately 0.3 μl each time) was tested at 3-day intervals. Furthermore, from the beginning of the experiment, changes in the body weights of the rats were also monitored once a week.

### Sample Collection and Preparation

At the end of the treatment period, the rats in the four groups were anesthetized *via* the intraperitoneal injection of 3% pentobarbital sodium (0.3 ml/100 g body weight), and blood samples were collected from the abdominal aorta using a vacuum tube, while liver tissue samples were collected from the abdominal cavity. The stools were collected from the cecum. The collected blood samples were then centrifuged at 4,000 rpm for 10 min at 4°C, and the supernatant was collected into liquid nitrogen, snap-frozen, and finally stored at −80°C until analysis. The stored serum samples were thawed at 4°C. The levels of different lipids in the serum samples were measured, including TC, TG, HDL-C, and LDL-C. Simultaneously, some of the collected hepatic tissue samples were used to test TC, TG, HDL-C, and LDL-C using the kits, while the other part of the hepatic tissue samples was fixed with paraformaldehyde for more than 24 h and, thereafter, used for hematoxylin-eosin (H&E) staining.

### Cell Line Culture and Cytotoxicity Assay

HepG2 cells were grown in DMEM containing 10% fetal bovine serum (FBS) and, thereafter, incubated at 37°C in a humidified atmosphere containing 5% CO_2_. Cells were passaged at a ratio of 1:3, and in the logarithmic growth phase, they were collected and used for the cytotoxicity assay experiments. The *in vitro* cytotoxicity of RGW was analyzed using a CCK8 assay kit (30). Briefly, the HepG2 cell suspension (1 × 10^5^ cells/ml) was inoculated into a 96-well cell culture plate at 100 μl per well, and after culturing for 24 h, the original medium was aspirated and different concentrations of the sample test solutions (0–400 μg/ml) were added to each well, and then incubated at 37°C for 72 h. Finally, 10 μl of CCK8 was added to each well 4 h before the end of the culturing process, and the optical density value was measured at 450 nm.

### Induction of Lipid Accumulation in HepG2 Cells, Drug Treatment, and Oil Red O Staining

Inoculated HepG2 cells (5 × 10^5^ cells/ml) were seeded into 6-well cell culture plates (2 ml/well). When the cells reached 10%–80% confluence, the culture medium was replaced with a serum-free medium and, after 12 h, oleic acid at different concentrations was added to induce the accumulation of intracellular lipids. Each concentration was repeated 3 times and incubated for 24 h.

Total ginsenosides of red ginseng were dissolved in the medium and assisted by adding 0.1% DMSO. The experiments at each concentration were performed in triplicate. Specifically, the cells were incubated with RGW at different concentrations (25, 50, and 100 μg/ml) for 24 h at 37°C; metformin (2 mM) was used as the positive control. Oil Red O staining was performed on treated HepG2 cells. The above cells were cultured in a 24-well culture plate with sterile coverslips, and the samples to be tested were added and cultured for 24 h. They were rinsed 3 times with PBS, fixed with paraformaldehyde for 30 min, rinsed with DW for 2 times, and stained with Oil Red O staining solution at room temperature for 30 min after rinsing, hematoxylin staining, and mounting. Finally, the results were observed under a microscope.

### Determination of Short-Chain Fatty Acids by Gas Chromatography-Mass Spectrometry

2-Ethylbutyric acid was prepared as a 0.1 mmol/L ether solution as an internal standard solution, and acetic acid, propionic acid, butyric acid, isovaleric acid, and valeric acid were prepared as 0.1 mmol/L standard solutions, which were used to prepare a standard curve at different concentrations.

A precision experiment was conducted as follows: the determination of the same sample solution was repeated 12 times, and the RSD value was calculated. A stability experiment was conducted as follows: a sample of treated stool was taken and it was measured two times at 0, 2, 4, and 6 h to calculate the RSD value. The standard recovery experiment was conducted as follows: a sample of rat feces was chosen after measurement, weighing 9 portions of 30 mg each. According to the concentration of the standard acid reference substance added to them, they were divided into three groups: high, medium, and low, with 3 copies in each group. The ratio of the concentration of the standard acid reference substance added to the previously determined acid concentrations in the high, medium, and low groups was 1.2:1, 1:1, and 0.8:1.

Furthermore, an appropriate amount of rat stool was dissolved in DW, vortexed for 1 min, and then centrifuged at 10,000 rpm for 10 min. Thereafter, 50% sulfuric acid solution and the internal standard solution were added, followed by vertexing for 1 min, and then centrifuged at 10,000 rpm for 10 min. The mixture was incubated at 4°C for 30 min, and the supernatant was collected for GC analysis. Chromatographic separation was then performed on an FFAP capillary column (30 m × 0.25 mm × 0.25 μm) using helium (1.0 ml/min) as the carrier gas. At a split ratio of 10:1, 1 μl of the sample solution was injected into the GC system, with the injector temperature set at 230°C, which was obtained using the following protocol: the initial temperature was maintained at 40°C for 2 min, and the temperature was increased at a heating rate of 10°C/min to 200°C for 5 min. Furthermore, the injection volume, transfer line temperature, and ion source temperature were 1.0 μl, 230°C, and 250°C, respectively.

### Statistical Analysis

All data have been tested for normality and were presented as mean ± SD and analyzed using Statistical Product and Service Solutions software (SPSS software). Student’s *t*-tests were used to compare the differences between the two groups. One-way analysis of variance (ANOVA) followed by Tukey’s multiple comparison test was used to compare more than two groups. *P* < 0.05 was considered to be statistically significant.

## Results

### UHPLC-MS Analysis of Red Ginseng

**FIGURE 1:**
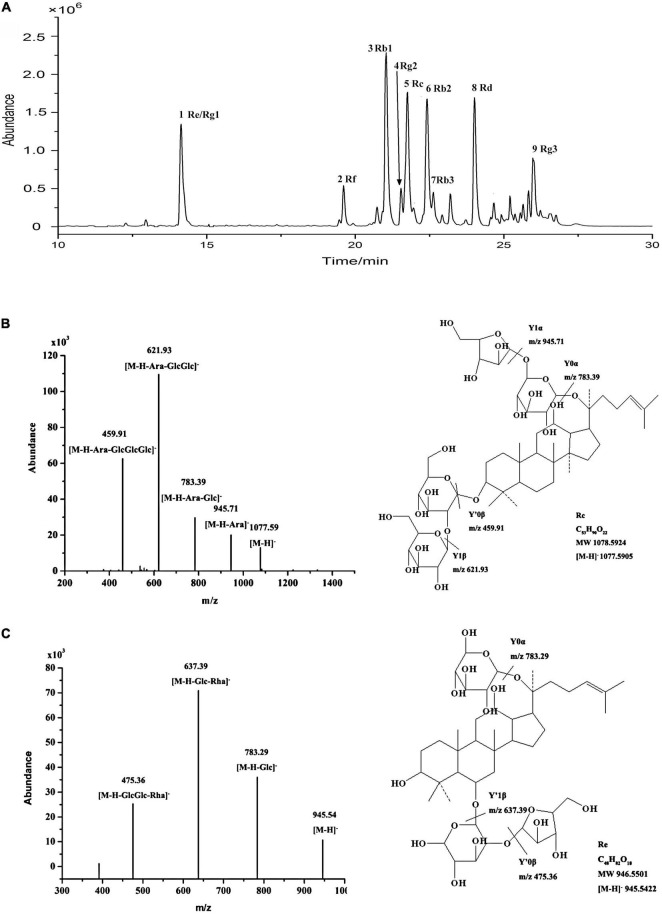
**(A)** UHPLC-Q-TOF-MS TIC of total ginsenoside of red ginseng. **(B)** ESI-Q-TOF-MS/MS spectra of ginsenoside Rc in negative ion mode. **(C)** ESI-Q-TOF-MS/MS spectra of ginsenoside Re in negative ion mode.

Using optimal chromatographic and MS conditions, a total of 10 major ginsenosides were identified in the obtained RGW. The presence of the ginsenosides, Re, Rg1, Rf, Rb1, Rg2, Rc, Rb2, Rb3, Rd, and Rg3 (Figure 1A), was confirmed by comparing their molecular weights and MS/MS (Figures 1B,C). In the negative ion mode, the ginsenosides appeared as the deprotonated [M-H]^–^ ion and [M + HCOO]^–^ ion. Negative ion mode was chosen for the following experiments, as it gives a much clearer fragmentation pattern for structural identification. The characteristic fragment ions of ginsenoside are summarized in Table 1. The characteristic product ions of aglycone PPD (m/z 459) and PPT (m/z 475) are observed. The types of sugar substitution were determined as hexose (glucose), deoxyhexose (rhamnose), and fructose (arabinose, xylose) with different linkages (Table 1). The characteristic neutral losses of these sugar residues were 162 Da (hexose), 146 Da (deoxyhexose), and 132 Da (fructose), respectively. Figures 1B,C shows the MS/MS spectra of representative types of ginsenoside PPD (Rc) and PPT (Re) as examples. In Figure 1B, the fragment ion at m/z 945 is produced by the loss of an arabinose residue of 132 Da. The ion at m/z 783 is generated by the loss of one arabinose-glucose residue (162 + 132 Da). The fragment ion at m/z 621 ion is generated by the loss of arabinose-glucose residue (162 + 132 Da) and glucose residue (162 Da). The ion at m/z 459 is produced by the loss of glucose–arabinose residue (162 + 132 Da) and glucose-glucose residue (162 + 162 Da), which is the characteristic ion of PPD-type aglycone. In Figure 1C, the ion at m/z 783 is generated by the loss of one glucose residue (162 Da). The fragment ion at m/z 637 ion is generated by the loss of glucose residue (162 Da) and rhamnose residue (146 Da). The ion at m/z 475 is produced by the loss of glucose-rhamnose residue (162 + 146 Da) and glucose residue (162 Da), which is the characteristic ion of PPT-type aglycone. The identified ginsenosides are shown in Table 1, which indicates that the mass accuracy for quasi-molecular ions and fragment ions was < 10 ppm, indicating that the detected molecular weights of the quasi-molecular and fragment ions were well matched with the corresponding theoretical values. To validate the quantitative analytical method, the linearity, regression, and linear ranges of the 10 ginsenosides were determined using the developed UHPLC-MS method. As shown in Table 2, the data indicated a good linear relationship between the investigated compound concentrations and their peak areas within the test ranges (*R*^2^ > 0.9979). Additionally, the overall RSD values of intraday and inter-day variations corresponding to the 10 ginsenosides were no more than 2.33% and 2.82%, respectively, and the results of our accuracy tests showed that the accuracy of the established method was also acceptable, with the overall spike recovery rates for the ginsenosides varying in the range from 98.75 to 105.23%. Regarding the stability of the method, the RSDs of the ten ginsenosides detected within 24 h were all below 2.23%. At signal-to-noise ratios (S/N) of 3 and 10, the lower limits of detection (LOD) and LOQ were less than 0.8 and 1.1 ng⋅ml^–1^, respectively. This validated LC-MS method was then applied for the quantitative determination of the 10 ginsenosides in RGW.

**TABLE 1:** Compounds identified from red ginseng (RGW).

			[M-H]^–^			Fragment ions of [M-H]^–^	
		
Peak	Identity	Molecular formula	Measd mass	Calcd mass	Mass accuracy (ppm)	MS (m/z)	MS/MS fragment ions
1	Re	C_48_H_82_O_18_	945.5428	945.5422	−6.77	945.5[M-H]^–^	783.29[M-H-Glc]^–^ 637.39[M-H-Glc-Rha]^–^ 475.36[M-H-GlcGlc-Rha]^–^
1	Rg1	C_42_H_72_O_14_	799.4849	799.4843	7.63	799.5[M-H]^–^	637.33[M-H-Glc]^–^ 475.99[M-H-GlcGlc]^–^ 392.61[M-H-GlcGlc-C_6_H_12_]^–^
2	Rf	C_42_H_72_O_14_	799.5206	799.5148	7.25	799.3[M-H]^–^	637.39[M-H-Glc]^–^ 475.28[M-H-GlcGlc]^–^ 391.17[M-H-GlcGlc-C_6_H_12_]^–^
3	Rb1	C_54_H_92_O_23_	1107.6048	1107.5951	8.76	1107.6[M-H]^–^	945.49[M-H-Glc]^–^ 783.36[M-H-GlcGlc]^–^ 621.53[M-H-GlcGlcGlc]^–^ 459.24[M-H-GlcGlcGlcGlc]^–^
4	Rg2	C_42_H_72_O_13_	783.4964	783.4894	8.93	783.6[M-H]^–^	637.51[M-H-Rha]^–^ 475.25[M-H-Rha-Glc]^–^ 391.17[M-H-Rha-Glc-C_6_H_12_]^–^
5	Rc	C_53_H_90_O_22_	1077.5905	1077.5845	5.57	1077.5[M-H]^–^	945.71[M-H-Ara]^–^ 783.39[M-H-Ara-Glc]^–^ 621.93[M-H-Ara-GlcGlc]^–^ 459.91[M-H-Ara-GlcGlcGlc]^–^
6	Rb2	C_53_H_90_O_22_	1077.5883	1077.5845	3.53	1077.5[M-H]^–^	945.65[M-H-Ara(p)]^–^ 783.57[M-H-Ara-Glc]^–^ 621.55[M-H-Ara-GlcGlc]^–^
7	Rb3	C_53_H_90_O_22_	1077.5830	1077.5845	4.18	1077.5[M-H]^–^	945.62[M-H-Xyl]^–^ 783.57[M-H-Xyl-Glc]^–^ 621.53[M-H-Xyl-GlcGlc]^–^ 293.18[XylGlc-H]^–^ 149.04[Xyl-H]^–^
8	Rd	C_48_H_82_O_18_	945.5361	945.5422	−6.45	945.5[M-H]^–^	783.46[M-H-Glc]^–^ 621.52[M-H-GlcGlc]^–^ 459.47[M-H-GlcGlcGlc]^–^ 161.14[Glc-H]^–^
9	Rg3	C_42_H_72_O_13_	783.4957	783.4894	8.04	783.6[M-H]^–^	621.70[M-H-Glc]^–^ 459.50[M-H-Glc-Glc]^–^

**TABLE 2:** Calibration curves, accuracy, precision, and content for 10 ginsenosides (*n* = 6).

Ginsenosides	Calibration curve	R^2^	Test range (μg)	Intra-day precision RSD (%)	Inter-day precision RSD (%)	Recovery (%)	RSD (%)	Content (mg/g)
Rb1	*y* = 0.2822x + 0.2156	0.9979	0.005–0.03	1.09	1.80	103.44	1.76	23.70 ± 0.08
Re	*y* = 0.2239x + 0.0552	0.9994	0.005–0.03	2.01	2.41	100.15	2.45	9.87 ± 0.32
Rf	*y* = 0.3001x + 0.1582	0.9982	0.005–0.03	2.09	2.06	98.89	1.88	8.01 ± 0.18
Rb2	*y* = 0.2648x + 0.1572	0.9990	0.005–0.03	1.75	0.99	105.23	2.25	6.78 ± 0.02
Rb3	y = 0.3437x + 0.1589	0.9992	0.005–0.03	2.14	2.12	101.20	1.87	2.59 ± 0.26
Rc	*y* = 0.2419x + 0.2927	0.9986	0.005–0.03	2.25	2.26	99.56	3.20	13.14 ± 0.07
Rd	*y* = 0.2355x + 0.1748	0.9992	0.005–0.03	2.33	2.57	102.50	1.23	9.46 ± 0.85
Rg3	*y* = 0.152x + 0.2133	0.9995	0.005–0.03	1.57	1.59	99.98	1.47	7.73 ± 0.47
Rg2	*y* = 0.2829x + 0.1751	0.9992	0.005–0.03	2.14	2.82	98.75	2.72	4.85 ± 0.39
Rg1	*y* = 0.2788x + 0.0981	0.9979	0.005–0.03	1.89	1.61	103.53	1.52	8.02 ± 1.21

*Data were expressed as mean ± SD.*

### Pathological Characteristics of Diabetic Mice

**FIGURE 2:**
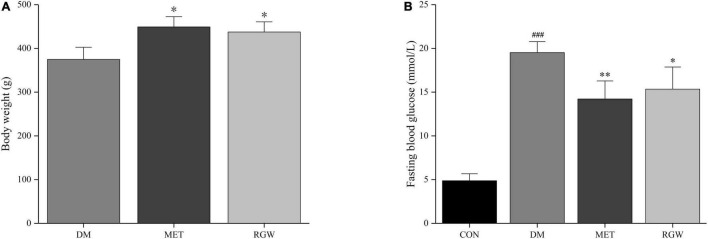
Pathological characteristics of diabetic rats. **(A)** Body weight. **(B)** Fasting blood glucose. Data are expressed as mean ± SD. *p < 0.05, **p < 0.01 vs. DM; ^###^*p* < 0.005 vs. CON (*n* = 8).

**FIGURE 3:**
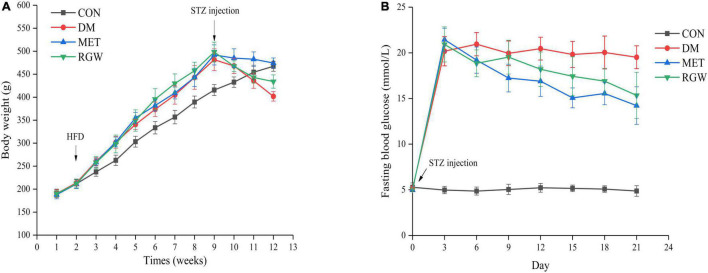
Effects of RGW on body weight and fasting blood glucose in DM rats. **(A)** Body weight. **(B)** Fasting blood glucose. Data are expressed as mean ± SD (*n* = 8).

After STZ administration, rats in the DM, MET, and RGW groups showed a significant increase in fasting blood glucose levels compared with those in the CON group (Figure 2B). Furthermore, STZ administration resulted in decreased body weight (Figures 2A, 3A), and symptoms of polydipsia, polyuria, and polyphagia were also observed. These findings demonstrate that the T2DM model was successfully established.

### Hypoglycemic and Hyperlipidemia Effects of Red Ginseng in T2DM Rats

The obvious characteristics of diabetes are a relative decrease in body weight gain and an increase in blood glucose levels. As shown in Figure 3A, the body weights of all the rats increased steadily, and their blood glucose levels were normal. However, the growth rate of the rats in the CON group was slower. Conversely, after STZ administration, the body weight of all rats began to decrease, and their blood glucose levels increased significantly relative to the rats in the CON group. Additionally, rats in both the MET and RGW groups tended to show a decrease in weight after STZ administration. The effect of RGW on the fasting blood glucose level of the rats is shown in Figure 3B, from which it is evident that the blood glucose levels of rats in the MET and RGW groups decreased gradually after 3 weeks of treatment. However, rats in the DM group only showed a slight decrease in blood glucose levels. At the end of treatment period, rats in both the MET and RGW groups showed significant decreases in fasting blood glucose level from 21.45 ± 1.25 mmol/L down to 14.22 ± 2.06 mmol/L (*p* < 0.01) and 20.92 ± 1.92 mmol/L to 15.34 ± 2.53 mmol/L (*p* < 0.05), respectively.

The lipid biochemical parameters of the T2DM rats in the different groups after treatment for 21 days are shown in Table 3. From this table, it is evident that rats in the DM group showed abnormal lipid metabolism as indicated by the increases in TC, TG, and LDL-C levels (*p* < 0.01) and a decrease in HDL-C levels (*p* < 0.01). However, after RGW treatment, serum TG, TC, and LDL-C levels decreased significantly (*p* < 0.01), while the level of HDL-C increased significantly (*p* < 0.01). Furthermore, the hepatic biochemical indicators showed abnormal lipid metabolism for rats in the DM group (Table 4). Further, hepatic tissues from rats in the RGW group also showed a significant increase in HDL-C (*p* < 0.01). The levels of TG and TC showed a downward trend, even though the decreases were not significant.

**TABLE 3:** Serum biochemical parameters of rats in different groups after being treated for 21 days (*n* = 8).

Items	CON	DM	MET	RGW
HDL-C (mmol/ml)	1.00 ± 0.13	0.65 ± 0.10**	1.29 ± 0.24^##^	1.16 ± 0.39^##^
LDL-C (mmol/ml)	0.56 ± 0.16	3.05 ± 0.50**	0.59 ± 0.16^##^	0.90 ± 0.54^##^
TG (mmol/ml)	0.54 ± 0.11	2.83 ± 0.19**	0.71 ± 0.12^##^	1.64 ± 0.65^#^
TC (mmol/ml)	7.49 ± 0.85	29.83 ± 6.17**	8.04 ± 0.32^##^	9.07 ± 3.31^##^

Data was expressed as mean ± SD. *p < 0.05, **p < 0.01 vs. CON; ^#^p < 0.05, ^##^p < 0.01 vs. DM.

**TABLE 4:** Hepatic biochemical parameters of rats in different groups after being treated for 21 days (*n* = 8).

Items	CON	DM	MET	RGW
HDL-C (mmol/ml)	0.27 ± 0.04	0.08 ± 0.01**	0.27 ± 0.05^#^	0.29 ± 0.05^##^
LDL-C (mmol/ml)	0.03 ± 0.01	0.32 ± 0.04**	0.02 ± 0.00^#^	0.04 ± 0.01^#^
TG (mmol/ml)	0.49 ± 0.11	0.48 ± 0.14	0.35 ± 0.03^#^	0.47 ± 0.12
TC (mmol/ml)	0.12 ± 0.02	0.14 ± 0.04	0.12 ± 0.01	0.11 ± 0.03

Data was expressed as mean ± SD. *p < 0.05, **p < 0.01 vs. CON; ^#^p < 0.05, ^##^p < 0.01 vs. DM.

### Pathological Changes in Hepatic Tissue of T2DM Rats

**FIGURE 4:**
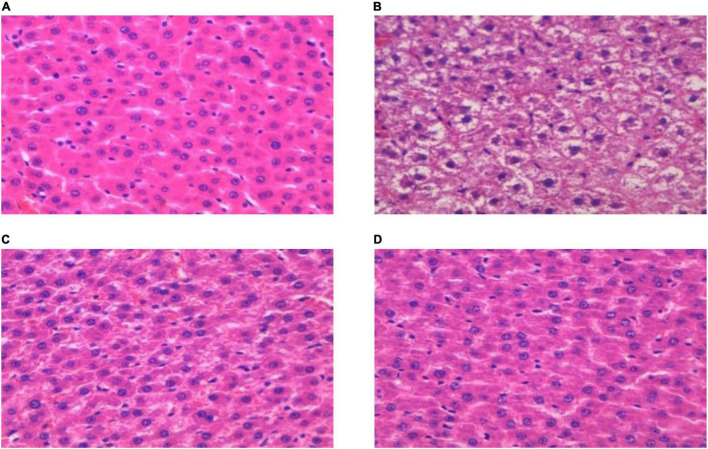
H&E staining of the hepatic (400 ×). **(A)** CON; **(B)** DM; **(C)** MET; and **(D)** RGW.

The histopathological characteristics of the hepatic tissue from each rat group were evaluated using H&E staining. The results thus obtained are shown in Figure 4, from which it is evident that in hepatic tissues from rats in the DM group, the cell arrangement was disordered (Figure 4A), with lipid droplet vacuoles of varying sizes and numbers present in the cytoplasm, and with most of the hepatocytes showing fatty degeneration (Figure 4B). But, after RGW treatment, the morphology of hepatic cells tended to be normal, and the size of fat droplets in the cells decreased significantly. Thus, RGW treatment almost completely reversed fatty degeneration in the hepatic cells (Figure 4D). Similar observations were made for the MET group.

### Effect of Red Ginseng on Lipid Metabolism

**FIGURE 5:**
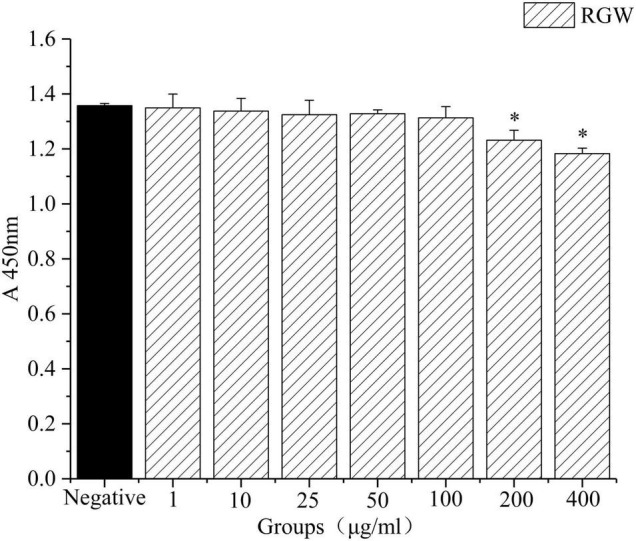
Effect of RGW on HepG2 cells. *p < 0.05 vs. negative.

**FIGURE 6:**
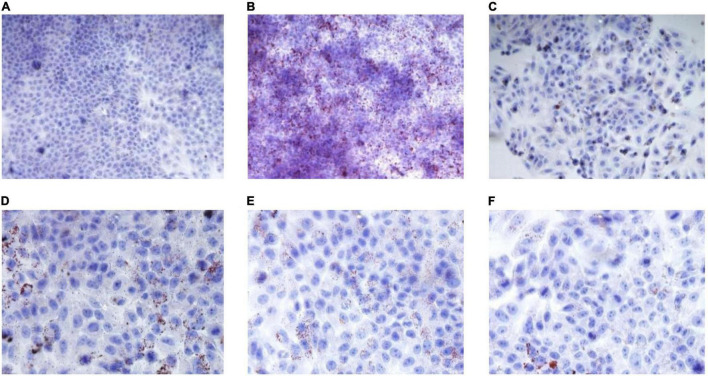
Effect of RGW on lipid metabolism. **(A)** Blank; **(B)** negative; **(C)** positive; **(D)** RGW 25 μg/ml; **(E)** RGW 50 μg/ml; and **(F)** RGW 100 μg/ml.

Oleic acid-induced HepG2 cells in a high-fat model of HepG2 cells were used to test the effect of RGW on lipid metabolism. After RGW treatment, cell viability did not decrease significantly at doses of 100 μg/ml as shown in Figure 5, showing lower cytotoxicity. Therefore, the maximum RGW dose was set at 100 μg/ml. As a fat-soluble dye, Oil Red O can specifically bind to triglycerides in tissues and cells to dye fat cells red. Thus, it was used to study the effect of RGW on lipid accumulation in HepG2 cells (Figure 6). After 24 h of incubation with oleic acid, a large number of red lipid droplets were observed in the cells, indicating that the lipid accumulation cell model had been successfully established. Our results further indicated that intracellular lipid accumulation decreased after RGW administration, and compared with the negative control group, the effect of the RGW treatment group was obvious, indicating that RGW could effectively inhibit lipid accumulation *in vitro*. Additionally, as the RGW dose increased, the sizes of the lipid droplets in the cells gradually decreased, and the inhibitory effect was more significant, highlighting an obvious dose-dependent manner.

### Determination of Short-Chain Fatty Acids

To validate the quantitative analytical method, the linearity, regression, and linear ranges of the 5 SCFAs were determined using the developed gas chromatography-mass spectrometry (GC-MS) method. The data indicated a good linear relationship between the investigated compound concentrations and their peak areas within the test ranges (*R*^2^ > 0.9950). Additionally, the overall RSD values of intraday and inter-day variations corresponding to the 5 SCFAs were no more than 3.66% and 3.72%, respectively, and the results of our accuracy tests showed that the accuracy of the established method was also acceptable, with the overall spike recovery rates for the five varying in the range from 92.44 to 113.95%. Regarding the stability of the method, the RSDs of the 5 SCFAs detected within 24 h were all below 4.04%. This validated GC-MS method was then applied for the quantitative determination of the 5 SCFAs.

As the end metabolites of the microbial fermentation of carbohydrates in the gut, SCFAs play an important role in lipid metabolism and participate in multiple lipid metabolism pathways. In this study, we examined the levels of five SCFAs based on the analysis of stool samples *via* GC-MS using the internal standard method. As shown in Table 5, compared with the CON group, the SCFA content of the stool samples from the rats in the DM group decreased significantly (*p* < 0.01). However, after RGW treatment, the levels of propionic acid, butyric acid, and isovaleric acid increased (*p* < 0.05). Those of acetic acid and valeric acid also increased, but the increases were not statistically significant.

**TABLE 5:** The content of five short-chain fatty acids (SCFAs) in rat stools (*n* = 8).

Items	CON	DM	MET	RGW
Acetic acid (umol/l)	347.76 ± 29.86	252.66 ± 15.49**	336.22 ± 19.15^#^	316.22 ± 22.79
Propionic acid (umol/l)	62.33 ± 7.40	27.79 ± 5.44**	48.12 ± 8.65^##^	51.16 ± 9.82^#^
Butyric acid (umol/l)	58.44 ± 15.86	35.37 ± 9.68**	57.36 ± 6.77^##^	43.38 ± 4.40^#^
Valeric acid (umol/l)	12.55 ± 2.09	6.52 ± 1.98**	10.84 ± 2.52^##^	9.60 ± 1.42
Isovaleric acid (umol/l)	14.10 ± 4.13	10.89 ± 1.92**	13.47 ± 2.53^##^	13.00 ± 0.74^#^

Data was expressed as mean ± SD. *p < 0.05, **p < 0.01 vs. CON; ^#^p < 0.05, ^##^p < 0.01 vs. DM.

## Discussion

In this study, a T2DM rat model was established to evaluate the regulatory effects of RGW on lipid metabolism disorders. The results demonstrated that RGW effectively improved lipid metabolism disorders caused by T2DM mainly through the lowering of blood lipids, reducing lipid deposition and increasing SCFAs levels.

Typical symptoms of T2DM include insulin resistance and abnormally elevated blood glucose levels. Particularly, insulin resistance plays a key role in the development of dyslipidemia in patients with T2DM, given that long-term insulin resistance in adipocytes leads to the increased production of free fatty acids, which in turn leads to an abnormal increase in TG levels (31). Our results showed that RGW treatment improved these parameters, leading to improved body weight as well as decreases in blood glucose and lipid levels during the intervention period. Thus, RGW has a regulatory effect on these metabolic disorders. Hyperglycemia is the main clinical manifestation of diabetes. This study showed that after 3 weeks of RGW treatment, rats in the DM group showed significant decreases in blood glucose levels, and those in the MET group showed a stronger hypoglycemic effect following this treatment. Thus, our experiments indicated that RGW has a good hypoglycemic effect, and this conclusion is consistent with those reported in some previous studies (32). Additionally, diabetes leads to the progressive accumulation of lipid metabolites, and the levels of TG, TC, LDL-C, and HDL-C are considered important biomarkers of hyperlipidemia (33). Our results confirmed that RGW showed a strong antihyperlipidemic effect by lowering TG, TC, and LDL-C levels while increasing HDL-C in T2DM rats.

The liver, as a central detoxifying organ of the body, plays a primary role in gluconeogenesis and lipogenesis regulation, and the abnormal physiological state of the liver is an important factor in the disorder of glucose and lipid metabolism (34). Experimental results indicated lipid deposition and cell degeneration in the liver of rats in the DM group, and this may have caused the abnormality of serum biochemical indicators. But owing to RGW treatment, this hepatic steatosis state was almost completely reversed. At the same time, the detection of liver biochemical indicators also verified this (Table 4). Lipid accumulation in the liver is caused by increased lipid acquisition and decreased lipid clearance. To verify how RGW ameliorated hepatic steatosis, experiments were performed using oleic acid-induced HepG2 cells. The experiments showed that the number of lipid droplets in HepG2 cells treated with 25, 50, and 100 μg/ml RGW decreased significantly, indicating that one of the reasons for the improvement in steatosis of hepatocytes may be a reduction in lipid deposition. Likewise, a study demonstrated that the reduction of hepatic steatosis leads to the recovery of liver function and the call-back of parameters related to lipid metabolism (35), which also confirmed our previous experimental results.

Short-chain fatty acids, which are produced *via* the fermentation of dietary fiber by gut microbiota, have beneficial health effects. However, their insufficient production is associated with T2DM and obesity (36). After 3 weeks of RGW intervention, increased SCFAs contents were observed. Reportedly, propionic acid and butyric acid contents are closely related to the incidence and prevention of T2DM. Administration of acetic acid has been shown to affect systemic lipolysis as well as intracellular lipolysis in adipocytes in *in vitro* and *in vivo* animal and human studies. (37). Additionally, G protein-coupled receptors (GPCRSs), which are SCFA receptors, including GPR43 and GPR41, play important roles in maintaining glucose and lipid metabolism. Specifically, GPR41 is activated by propionate to increase insulin sensitivity and maintain energy and glucose homeostasis, while GPR43 can be activated by acetate in white adipose tissue to modulate energy uptake and improve glucose and lipid metabolism (38). In addition to acting as signal molecules, SCFAs can also serve as energy substrates in adipocytes. Furthermore, in hepatocytes, acetate and butyrate are mainly involved in lipid biosynthesis, whereas propionate is primarily involved in gluconeogenesis, and in adipocytes, SCFAs may enter the adipogenic pathway after being absorbed and activated by short-chain coenzyme synthase (39). It has also been observed that propionate is an important factor that influences hepatic gluconeogenesis and lipid metabolism (40, 41). This study confirms the importance of propionate to inhibit hepatic lipogenesis and improve insulin sensitivity in high-fat diet-induced obesity (42). Another factor in the reversal of hepatocyte steatosis and lowering of blood glucose levels by RGW may be mediated in part by the effect of propionate on hepatic carbohydrate metabolism. Additionally, butyrate, which is the main energy source for colonic epithelium, can induce gluconeogenesis in the intestine, thereby improving glucose and energy homeostasis. Additionally, it can enhance fatty acid oxidation and energy consumption in the human body (43). Thus, the increase in butyrate content following RGW treatment may also be responsible for the decrease in blood lipid and glucose levels in diabetic rats.

In this study, the RGW were quantitatively analyzed using UHPLC-MS and its effect on the lipid metabolism of T2DM was studied. We observed that after 21 days of RGW treatment, all the diabetes-related parameters corresponding to the T2DM rats improved. Moreover, *in vitro* cell experiments demonstrated that RGW had a significant lipid deposition improvement effect. These experimental results provide a usable basis for functional food development for the treatment of diabetes. Additionally, our results imply that RGW, which is easy to obtain and has controllable quality, can be developed as a potential natural antidiabetic food for the prevention and treatment of obesity-related diabetes.

## Data Availability Statement

The original contributions presented in the study are included in the article/supplementary material, further inquiries can be directed to the corresponding author.

## Ethics Statement

The animal experiment was approved by the Institutional Animal Care and Use Committee (IACUC) of Changchun University of Chinese Medicine.

## Author Contributions

RH: methodology, investigation, and original draft preparation. YTo, MZ, YTe, and HL: validation and software. WW: conceptualization, methodology, supervision, and funding acquisition. All authors contributed to the article and approved the submitted version.

## Conflict of Interest

The authors declare that the research was conducted in the absence of any commercial or financial relationships that could be construed as a potential conflict of interest.

## Publisher’s Note

All claims expressed in this article are solely those of the authors and do not necessarily represent those of their affiliated organizations, or those of the publisher, the editors and the reviewers. Any product that may be evaluated in this article, or claim that may be made by its manufacturer, is not guaranteed or endorsed by the publisher.
